# Complexities and challenges of translating intervention success to real world gait in people with Parkinson’s disease

**DOI:** 10.3389/fneur.2024.1455692

**Published:** 2024-10-09

**Authors:** Charlotte Lang, Jaap H. van Dieen, Matthew A. Brodie, Julius Welzel, Walter Maetzler, Navrag B. Singh, Deepak K. Ravi

**Affiliations:** ^1^Laboratory for Movement Biomechanics, Institute for Biomechanics Department of Health Science and Technology, ETH Zürich, Zürich, Switzerland; ^2^Department of Human Movement Sciences, Faculty of Behavioural and Movement Sciences, Vrije Universiteit Amsterdam, Amsterdam Movement Sciences, Amsterdam, Netherlands; ^3^Graduate School of Biomedical Engineering, University of New South Wales, Sydney, NSW, Australia; ^4^Department of Neurology, University Medical Center Schleswig-Holstein, Kiel, Germany; ^5^Singapore-ETH Centre, Future Health Technologies Program, CREATE Campus, Singapore, Singapore

**Keywords:** Parkinson’s disease, gait, intervention, free-living, laboratory

## Abstract

**Background:**

Unstable gait leading to falls negatively impacts the quality of life in many people with Parkinson’s disease (PD). Systematic review evidence provides moderate to strong evidence of efficacy for a wide range of physiotherapy-based interventions to reduce gait impairment. However, outcomes have often focused on gait assessments conducted in controlled laboratory or clinical environments.

**Objective:**

This perspective investigates the complexities and challenges of conducting real-world gait assessments in people with PD and the factors that may influence the translation from improved lab-assessed gait to improved real-world gait.

**Methods:**

Through a thorough review of current literature, we present an in-depth analysis of current methodological approaches to real-world gait assessments and the challenges that may influence the translation of an intervention’s success from lab-based outcomes to improved walking during daily life.

**Results:**

We identified six key factors that may influence the translation of intervention success into real-world environments at different stages of the process. These factors comprise the gait intervention, parameters analyzed, sensor setup, assessment protocols, characteristics of walking bouts, and medication status. We provide recommendations for each factor based on our synthesis of current literature.

**Conclusion:**

This perspective emphasizes the importance of measuring intervention success outside of the laboratory environment using real-world gait assessments. Our findings support the need for future studies to bridge the gap between proven efficacy for gait as assessed in controlled laboratory environments and real-world impact for people with PD.

## 1 Introduction

Gait impairments are among the most common and disabling symptoms in Parkinson’s disease (PD) as they limit mobility and often lead to falls, reducing the quality of life (1). Approximately 60% of people with PD fall at least once per year (2). An estimated 50% of all falls in people with PD occur during walking, often caused by inability to adapt to environmental demands including perturbations or disturbances (3, 4). Following years of extensive research, we now have a robust understanding that gait impairments in PD are commonly characterized by reduced gait speed, shorter step length, longer double-support phase, higher stride-to-stride variability, and more steps to complete a turn (5, 6). Furthermore, the severity of these gait impairments increases as the disease advances leading to adverse mobility outcomes such as freezing of gait, that further exacerbates the risk of falling (7, 8).

A broad range of non-pharmacological interventions with the goal of managing motor symptoms, enhancing gait quality, and consequently, mitigating the risk of falls are available, including treadmill and overground gait training, resistance exercises, and other complementary therapies such as Tai Chi (9). A recent meta-analysis that included 191 trials generally found positive evidence supporting all these interventions in the management of PD (10). Among them, treadmill training emerged as the intervention with the most compelling evidence for improving gait parameters such as speed (18 out of 22 trials) and step length (14 out of 17). The ‘efficacy’ of such interventions is normally evaluated by the assessment of gait changes in gait parameters of interest inside the laboratory using motion capture systems (9–11). However, it is crucial to extend the assessment beyond the controlled laboratory to evaluate the ‘effectiveness’ of an intervention in broader, ecological, real-world settings. If an intervention is efficacious inside the laboratory, but proves ineffective outside of its controlled environment, consideration needs to be given to modifying existing gait interventions or exploring new ones.

Recent advances in wearable movement sensors have made the assessment of gait outside of the laboratory feasible. Several studies have compared walking performance between laboratory settings and daily life (12–14). These investigations have revealed a disparity between gait observed in laboratory settings and that in real-world scenarios, likely stemming from the intricate interactions we encounter in everyday life, resulting in reduced attention toward gait itself (12, 13, 15). Although gait is different in the real-world for healthy individuals, such differences are more pronounced in people with PD (13). In daily life, patients with PD can exhibit a reduction in gait speed by about 30% compared to laboratory settings, while differences in stride length can be around 20% (13). Moreover, in the real-world scenario, gait is constantly challenged by factors such as turns, stairs, or obstacles among others (15). In addition, variability and asymmetry in daily life gait may be evoked by, e.g., diverse furniture or a busy environment. Distractions and multitasking situations further influence these gait characteristics. Hence, evaluating gait in a laboratory setting can provide insights mostly into an individual’s capacity (what a person can do), while observing gait during daily life most likely offers a reflection of a person’s functional performance (what and how a person does) (16). This implies that assessments are required both in the laboratory and in real-world settings, as the information they provide is complementary and both must be taken into account for treatment decisions in PD.

The extent that interventions aimed at improving key indicators of PD disease severity in a laboratory translate to improvements in gait quality and quantity in daily life remains uncertain. In this context, ‘translation’ refers to the process of effectively transferring outcomes or findings observed in a controlled laboratory setting to real-world or daily life scenarios. It implies assessing whether (and by how much) the positive effects of interventions observed in a laboratory environment can effectively impact on the ‘same’ gait parameters or mechanisms in the real-world. In this understanding, no current intervention literature fits this scope, but a few papers have attempted to measure both laboratory and real-life gait. Rieger and coauthors report improved gait performance in a laboratory setting after a 4-week perturbation-based treadmill training with dual-tasks for healthy older adults, but no changes in daily-life gait quality or quantity (17). In people with PD, only one study has examined the effectiveness in terms of improvements in gait quality in real world contexts (18). The study by Cohen and coworkers found improvements in laboratory-based tests that are intended to reflect walking abilities (6MWT, 10MWT and TUG), after an 8-week multidisciplinary intensive outpatient rehabilitation program for PD patients. Real-world gait quantity (e.g., step count) or quality (e.g., gait speed, step length), however, did not show any (18). Another study will assess the effects of a turning intervention on clinical measures and on turning and gait quality in real-life among people with PD (19). Furthermore, many studies only report on daily life step count or the number of falls in daily life to evaluate effects after an intervention, while quality of gait is not considered (20–23). This raises the question whether we can anticipate a direct translation of the effects by training interventions for people with PD within the laboratory to gait in the real world. Psychological, physiological, pathological, cognitive, environmental, and technical factors influence the outcomes and may cause a disparity between laboratory assessments and real-world environments (15). Even though gait measures taken in a laboratory setting may not reflect those observed in daily life, there appears to be some evidence indicating that certain gait parameters, such as gait speed, exhibit similar associations between clinical and real-world settings in both PD patients and healthy controls (24). Also gait parameters derived from standardized tests in clinical and free-living settings seem to be related (25).

Given the significant impact of gait impairments on daily-life walking, achieving the translation from laboratory assessments to real-world gait through intervention is essential for enhancing quality of life and reducing the incidence of falls in people with PD. Addressing the challenge of successful translation is crucial for unraveling this real-world complexity and enhancing our ability to assess intervention effects in daily life. However, it might be due to the complexity of the real world, and thus the complexity of outcomes, that gait assessment remains often restricted to the laboratory, while it is refrained from attempting measurements in daily life. To achieve these translational goals, optimize existing treatments and increase intervention effectiveness, more real-world research in people with PD is required. This narrative review is offering a window into real-world complexity of gait assessment, particularly concerning the translation of intervention effects in PD. We provide approaches to navigate and address these complexities by identifying aspects that influence real-world assessment and may support researchers in managing real-world data. In this context, our focus was primarily on non-pharmacological interventions for gait and the translation of their effects to real-world settings. The search terms encompassed, but were not limited to, “real-world,” “daily life,” “Parkinson’s disease,” “intervention,” “rehabilitation,” “IMU,” “wearable sensor,” and “gait.” Given the limited number of real-world studies specifically focused on individuals with Parkinson’s disease, research involving healthy adults or stroke patients was also included. In particular, we focus on six factors that might have an influence on the translation of intervention effects in PD to the real world at different stages of the process: the gait intervention, analyzed parameters, number and placement of IMU sensors, assessment protocol, walking bouts and medication status. By identifying the conditions for each factor where the disparity between laboratory and real-world will be minimal and performing gait interventions under representative conditions as well as adapting the protocol for real-world assessments, we may be able to facilitate a translation. Achieving a direct translation could help understanding the effects of an intervention in daily life and lead to an optimization of current interventions in PD.

## 2 Factors influencing translation of gait outcomes in PD

**Figure 1:**
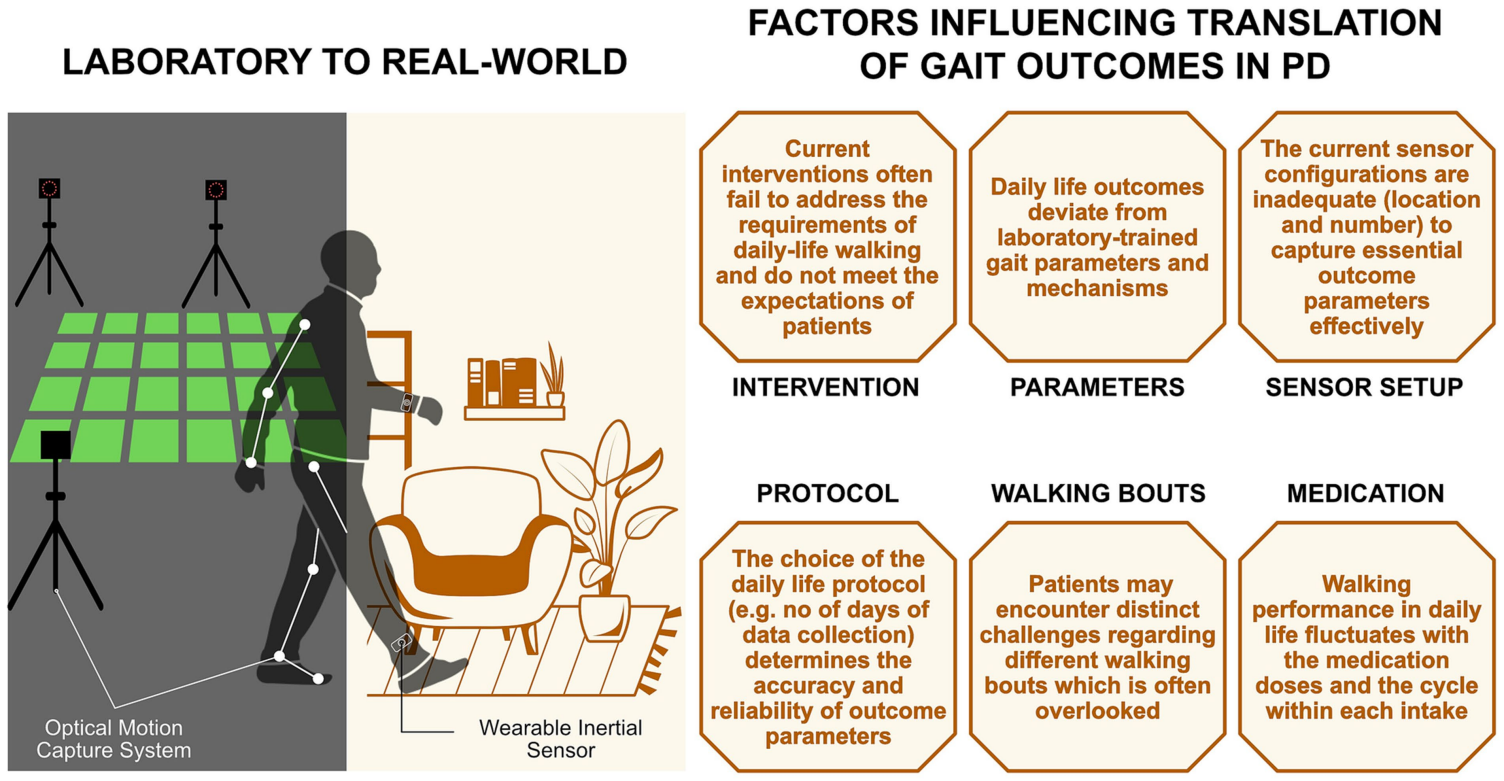
Factors influencing the translation of gait outcomes from the laboratory to the real-world in people with PD.

To bridge the gap between laboratory and real-world assessments, we identified and discuss six factors (Figure 1), addressing which may aid in achieving translation of gait interventions in PD.

### 2.1 Intervention

A large number of studies investigated the effects of treadmill training on gait parameters, therefore having the largest evidence of positive effects (26–28). Treadmill training is a form of externally cued gait training that utilizes somatosensory cues through movement and speed to encourage stepping (26). Different types of treadmill training are frequently used, for example speed-dependent treadmill training with short intervals of fast speeds or treadmill training with progressive speed development over time. There is some evidence that treadmill training has (short-term) effects on gait parameters including gait speed, stride length, cadence or walking distance (26, 29) and PD patients seem to be able to transfer effects to overground walking in a laboratory (30). Treadmill training further seems to provide long-term effects for gait performance in the laboratory of three to 6 months (5). However, these effects might not provide practical benefits in daily life, as treadmill training does not reflect typical demands of a natural environment, which seems to be an important factor for translation of gait improvements in the laboratory to the real-world. Therefore, the effectiveness of treadmill training in translating benefits to real-world gait and other physical functions is yet to be determined. Various concepts were developed with the goal to mimic real-world situations, including virtual reality, obstacle crossing or external perturbations (31–33). Adding dual tasks, like a visual oddball or an auditory Stroop task while walking in a virtual reality simulation of the real world, mimics daily gait more closely (34, 35), as everyday activities often involve motor-cognitive challenges and put demand on executive processes, e.g., when walking while talking to others or carrying an object. Therefore, including dual tasks in the intervention might induce a better translation of intervention effects to real-world gait. Moreover, evidence suggests that task-specific training is necessary to achieve the largest training effects (9, 36). However, task-specificity may not be applicable to various real-world situations, where walking conditions constantly vary, potentially leading to less transferability to real-world gait (37).

### 2.2 Parameters

Most commonly, gait speed, step or stride length, and cadence are used as outcome parameters for evaluating the efficacy of an intervention in a laboratory (24). Here, these parameters frequently improve after an intervention (8, 24). While spatiotemporal parameters such as stride length variability or asymmetries are also employed, their utilization in daily life is less common. When evaluating the effectiveness in real-world settings several studies used questionnaires to evaluate quality of life or number of falls or investigate daily step count or walking time following an intervention. When only looking at studies calculating walking-related digital mobility outcomes, similar to the laboratory, gait speed is commonly evaluated, as it is often considered as the “final common expression” of locomotor control (38, 39). However, gait speed does not fully capture the underlying mechanisms governing locomotion (40). Therefore, as a first step, it is important to ascertain which specific gait parameter(s) the intervention is targeting directly. This gait parameter thus becomes the suitable outcome parameter for evaluating the efficacy (improvements in the laboratory) and effectiveness (improvements in daily life) of the intervention, thereby facilitating translation from laboratory settings to real-world (41).

In addition to the previously validated digital mobility outcomes, like gait speed or step length, that are related to future disease progression, falls, physical function, and cognition (24), further parameters need to be explored for application in real-world settings. Examining parameters that are less affected by environmental factors might be beneficial for observing and assessing translation. In this respect, a study involving young healthy adults showed that different settings (laboratory or real life) and testing conditions influence gait variability parameters, whereas gait stability parameters such as Sample Entropy, remain unaffected by such variations (42). Furthermore, in older healthy adults, Sample Entropy seems to be only weakly affected by walking speed (43). Therefore, including parameters less susceptible to environmental or other influences could prove beneficial for assessing translation. However, future studies, particularly focusing on individuals with PD, are necessary to comprehensively understand the potential benefits.

### 2.3 Sensors (number/placement)

Evaluating an individual’s gait quality in real-world conditions requires mobile sensors that are able to capture the relevant outcome parameter while also being worn comfortably by the patient over several days. However, the accuracy and reliability of gait parameters depend on the sensor setup, including the number of sensors and their position on the body. Algorithms need to be validated and improved, which is often still done in a laboratory with a rather low complexity of gait tasks and restricted observation period (44, 45). In the real-world, additional aspects such as gait speed and contextual factors like stairs or turns and use of walking aids may alter the gait pattern and influence the performance of algorithms used for calculating gait events (15). This further challenges the analysis of real-world data and undermines the reliable determination of gait parameters.

In previous studies, different IMU configurations were utilized, ranging from a single sensor to setups with as many as 11 sensors (46–50). Frequently used sensor locations include lower back, feet, wrists, or shanks, with wrist, ankle and lumbar sensors exhibiting high wearability among individuals with PD (50). When employing multiple sensors, there is still a lack of agreement on the ‘optimal’ setup, leading to many possible combinations (51). Currently, the most commonly used setup consists of a single sensor on the lower back (51). The assessment of spatio-temporal gait parameters requires the accurate detection of initial and final contact of the foot, which is essential for further determination of spatio-temporal gait parameters. Gait events can be reliably detected using only one sensor on the lower back, demonstrating accurate identification of initial contacts in both healthy individuals as well as in people with PD (52, 53). However, determination of the final foot contact using a lower back sensor is less accurate compared to shank-worn sensors, which provide higher accuracy due to their closer proximity to the point of contact between foot and ground (54–56). Consequently, the estimation of spatial parameters (e.g., step length) still seems to be challenging and presents higher errors than evaluated from sensors on the shank. This becomes even more important as errors in the estimated step length introduce errors in the estimation of gait speed (57). Furthermore, for measuring step-to-step variability, the accurate detection of gait events is crucial. Motor asymmetries often are the first motor symptoms of PD that tend to decrease with disease progression (8, 58). Assessing asymmetry with just one sensor can be challenging, while this can be accurately estimated with two sensors on the feet or shanks (59, 60). However, parameters such as the harmonic ratio, that try to estimate the symmetry of gait patterns, have been employed in real-world measurements with a single sensor (61, 62). Especially in challenging real-world situations such as turns or stair climbing, sensors placed on the shanks or feet currently provide reliable differentiation of left and right gait events. As current interventions likely only lead to small effects in real-life, it is critical to estimate gait parameters as accurately and reliably as possible (63). Therefore, including a sensor at the foot level in addition to a sensor at the lower back in the assessment of real-world gait might be beneficial in facilitating translation and enable the calculation of further relevant parameters such as foot clearance. Alternative wearable sensors, aside from IMUs, could also serve this purpose effectively. For instance, plantar pressure insoles offer accurate and reliable data on gait events (55, 64), while infrared distance sensors can detect the alternating movements of the lower extremities (65).

Nevertheless, the acceptability of a 3-sensor system needs to be considered as adherence by PD patients might be low. Achieving a high adherence requires the setup to be as imperceptible as possible, and the benefits should be communicated as they might not be directly perceivable by patients. Still, currently high quality and accuracy of data might outweigh the eventually perceived burden as many aspects and mechanisms in PD, especially in daily life, are still unknown and highly successful interventions are crucial to improve patients quality of life. Future studies should also add questionnaires about usability and wearability of the sensors to gather patient experience. This approach would facilitate the evaluation and enhancement of current sensor setups. This includes not only the number and placement of sensors but also factors such as wearing comfort, practicality in conditions such as warm or humid environments, the adjustability of sensor attachments, and participant requirements, such as removing and reattaching sensors for charging.

### 2.4 Protocol

Extensive research in the last years has led to rapid developments in the field of wearable devices and enables collecting data continuously over several days or weeks. Nevertheless, the duration required to reliably assess both movement characteristics in everyday physical activity and their day-to-day variability is still unknown (66–68). Patients with PD exhibit high day-to-day variability of performance measures such as steps per day (69). Therefore, it is necessary to collect data over multiple days to provide an accurate representation of gait characteristics (70). Furthermore, the amount of activity may vary between weekends and weekdays and could be influenced by weather conditions (71, 72). The number of days for data recording currently varies between three and 7 days (12, 47, 73–76). In healthy older adults, two consecutive days can already reliably assess many activities (e.g., sitting, standing) as well as parameters (e.g., number and length of walking bouts). However, the median walking bout duration requires 5 days of measurement to remain reliable (66). A minimum of 3 days may be sufficient in healthy older adults to ensure a reliable and valid assessment. However, in people with PD, three up to 7 days of recording are required to reliably estimate certain measures, such as symmetries, using a single sensor on the lower back (77). Given their increased day-to-day and within-day variability as well as reduced physical activity (78), longer recording durations are needed compared to healthy adults to ensure reliable assessment of different gait measures, potentially as long as it can be integrated with participants wear time compliance. In line with that, the recording should last five consecutive days of daily living, however, a minimum of 3 days is essential to ensure a reliable and valid assessment. Moreover, maintaining similar settings during pre-and post intervention assessment, while allowing participants to engage in their usual activities without restriction, is important. This involves having participants reside in the same environment for both measurements, preferably at their own homes. A questionnaire could be used to gather crucial information about any specific activities that the patient may engage in within or outside the home that might vary between the two assessments. Additionally, it could provide insight into the utilization of assistive devices such as walking aids. The influence of psychological and motivational aspects on daily activity might be difficult to collect objectively but needs to be considered when analyzing real-world gait data. Fear of falling often leads to a reduction in physical activity, resulting in reduced confidence, further increasing fall risk and reducing quality of life (79, 80). In addition, people with PD with low self-efficacy are less likely to participate in physical activity (81). On the other hand, gait impairments might be improved due to increased levels of motivation, as a result of switching to a goal-directed mode of control, e.g., when participating in a study (82). Therefore, a comprehensive understanding of these aspects could lead to more effectively customized interventions that address not only physical impairments but also the motivational and psychological barriers to mobility.

### 2.5 Walking bouts

Another important aspect is the dependency of reliability of gait parameters on the length of the walking bouts analyzed in daily life. A walking bout is defined as a period of continuous walking, but variations in the criteria for the number of consecutive steps/strides used to define a walking bout across studies make comparisons challenging (73, 83, 84).

In daily life measurements, most walking bouts have a duration shorter than 10s or fewer than 12 strides (12, 84). However, according to Del Din et al. and Rehman et al., shorter walking bouts do not discriminate between people with PD and healthy people and therefore, longer walking bouts (>30 s) should be investigated when evaluating daily life gait characteristics (12, 85). However, in their study, only around 12% of all walking bouts were longer than 30s (85). Therefore, only investigating those bout lengths would not give a complete picture of the actual walking performance. In contrast, Shah et al. found that differences between those two groups were more apparent in shorter bouts (84). The difference between the results might be explained by the utilization of different sensor setups in these studies: a single sensor on the lower back versus three sensors on the feet and the lower back. Sensors on the feet could enhance the accuracy of detection for short walking bouts (86). Nevertheless, to reliably capture gait variability measures in a laboratory, a minimum of 50 gait cycles is necessary (87). This is supported for real-world assessments by the findings of Micó-Amigo co-workers, who reported larger errors in gait event detection in walking bouts with a duration of less than 8 s using a single sensor at the lower back, which is crucial for a reliable estimation of gait variability (52). As gait parameters like gait speed or stride length increase with increasing bout lengths (84), including all gait bouts in the analysis, but separate them and compute gait parameters according to the walking bout length might provide additional information about gait characteristics in real life.

### 2.6 Medication status

Unsupervised measurements in daily life encompass all phases of the medication cycle, including ON and OFF phases. Due to greater regimen complexity with increasing number of daily dose intakes and cognitive deficits, medication adherence becomes more challenging in people with PD (88, 89). In addition, it has been shown that walking patterns differ when measured during peak dose (at least 30 min after medication intake) or at the end of the dose (90). To the contrary, interventions in the laboratory are usually performed during (peak) ON medication. Therefore, determining average gait parameters throughout an entire day does not provide comparable results, making it challenging to evaluate the translation and effectiveness of an intervention. To adequately quantify the impact of the medication state, successfully translating the effects may entail observing the patient under similar conditions during both unsupervised assessment and intervention phases. Additionally, during pre-and post-assessments, variations in medication intake must be accounted for, as the dosage may differ between assessment days. Documenting times of medication intake, e.g., by using a diary or tracking with a smartphone app, would facilitate the identification of phases with similar medication levels (91). However, despite being the current standard for assessing medication intake and motor fluctuations, diaries may not be feasible for reliable documentation (92). Instead, current implementations involve the monitoring of medication adherence using a smartwatch, where patients receive reminders for their medication intake times and can either confirm or decline the intake, or utilizing machine learning techniques to differentiate between ON and OFF phases (93, 94). Another successful approach could be measuring the optimum state during the real-life assessment, including the best 10% of an individual’s distribution (15). Both approaches would facilitate the comparison of gait patterns at equivalent mobility levels and minimize the influence of medication on gait outcomes. Moreover, this allows tracking to identify in which phase of the medication cycle interventions can improve gait outcomes and may lead to a refinement of interventions, enabling improvement in gait patterns throughout the day.

## 3 Conclusion

Despite the growing evidence that gait interventions in laboratory settings can improve gait among individuals with PD within that controlled environment, it remains uncertain whether these effects translate to real-world scenarios. In this context, we propose defining ‘translation’ as ‘the process of effectively transferring outcomes or findings observed in a controlled laboratory setting to the ‘same’ gait parameters or mechanisms in real-world or daily life scenarios.’ Achieving a translation is important to improve current gait interventions and evaluate their real-world effectiveness. Currently, only a limited number of studies investigate intervention effects in both laboratory and real-world settings. Alongside the previously mentioned intervention studies involving individuals with PD and healthy adults, further evidence exists in multiple sclerosis (MS). A multidisciplinary inpatient rehabilitation program for people with MS has shown similar outcomes, improving gait capacity in the laboratory but not translating to enhanced gait performance in daily life (95). The authors proposed incorporating behavioral and psychological factors, such as motivation and self-efficacy, into rehabilitation programs, which may contribute to achieving long-term effects with increased physical activity. In addition to these factors, disease severity and environmental challenges also need to be accounted for while motivating individuals toward increasing the level of participation in physical activity.

Furthermore, none of these studies align with our definition of translation. Instead of clearly defining which gait mechanisms and corresponding gait parameters the intervention targets, and then analyzing them in both laboratory and real-world settings, no direct comparison or analysis of the same gait outcomes has been made. Most commonly, real-world evaluations following an intervention have focused on gait speed, fall occurrences, or activity level measured by steps per day. Gait speed is often considered as the “final common expression” of locomotor control, while falls and physical activity are closely linked to quality of life. Consequently, these metrics are typically viewed as the ultimate indicators of intervention success. However, improvements in these “final outcomes” are often the result of changes in specific underlying gait mechanisms or gait quality. To date, it remains unclear whether the same gait mechanisms are involved in both laboratory and daily life settings. Gait interventions are effective because they target and improve specific mechanisms through training. Therefore, it is essential to evaluate both the direct translation of these improvements and the underlying mechanisms that facilitate the translation of intervention effects to daily life. This is particularly important in PD, where motor learning processes and the ability to transfer skills to different tasks may be impaired. Understanding this relationship is crucial for optimizing gait interventions and ensuring their long-term effectiveness in improving daily functional outcomes. As there still seems to be reluctance toward real-world assessment, potentially due to the challenges posed by the complexity of the environment and the multitude of influencing factors, we provide potential solutions to unravel the real-world complexity (Table 1). By that, we aim to provide the basis from which concrete recommendations will emerge, guiding best practices in this area.

**Table 1:** Challenges and potential solutions to unravel real-world complexity and advance the application of daily life assessment in the evaluation of gait interventions in PD.

	Current challenges	Potential solution
Intervention	Current interventions do not reflect typical demands of the natural environment, potentially diminishing their translational efficiency	Mimic real-world environment more closely by adding dual-task, perturbation or AR/VR environments
Parameters	Assessment of different gait outcomes (clinical gait tests vs. gait quality) in both laboratory and real-world settings complicates the observation of translation and understanding of underlying mechanisms	Determine which parameter is targeted by the intervention to monitor changes in both laboratory and real-world settings and evaluate if similar mechanisms apply in both settings
Sensor setup	The assessment with IMUs needs to find a balance between an accurate and reliable assessment of gait parameters while being comfortable for the participant	Development of a 3-sensor setup with sensors on the ankles that ensures patient comfort while accurately evaluating gait quality
Protocol	Variations between home assessment time points hinder the comparison of pre-and post-assessment The duration for a reliable assessment of gait parameters is still unknown	Ensure consistency in the environment for both assessments. Documentation of additional information about activities to gain contextual information
Walking bouts	Different walking bout lengths are included in the analysis and lead to varying results	Identify meaningful clusters of walking bouts and include them all into analysis
Medication	Changes of gait pattern with medication cycle and their influence on translation are not considered	Times of medication intake should be documented to observe the influence of the medication cycle on gait and intervention effects. Passive methods for documentation need to be explored to reduce patient involvement

By identifying the factors influencing real-world assessment in PD, this paper proposes possible solutions to mitigate the disparities between laboratory and real-world settings, particularly for gait interventions in PD and their translation to real-life. This might allow to improve our understanding of current gait interventions, their effectiveness in the real world and the optimization of these treatments. However, it is important to recognize that for patients, translation or improvement of gait parameters, such as gait speed, may hold different significance. For them, the objective might rather entail improvements like being able to walk to the store. Hence, it is essential to also consider patient reported outcomes and connect them to gait parameters to obtain a comprehensive understanding of translation and real-world effectiveness. In lab-based assessments, minimal clinically important differences are established to indicate the meaningful impact of an intervention. However, these thresholds still require reassessment in real-world environments. Certain gait parameters, like stride time variability, show substantial increases in real-world settings compared to laboratory environments (e.g., treadmill assessments) (61). These discrepancies must be considered when assessing the effectiveness of an intervention. To identify a meaningful change, one approach might be to compare the distributions of data collected in and outside the lab, while adjusting for differences in their respective standard errors. Alternatively, combining real-world gait outcomes with patient-reported outcomes may signify a meaningful change if improvements in gait outcomes can also be perceived by the patient. By exploring this in future studies and examining how changes in gait outcomes impact quality of life and activities of daily living, it may become possible to connect subjective patient reports with objective gait data, thereby improving effectiveness through a more patient-centered approach.
